# Khat Chewing Practice and Associated Factors among Adults in Ethiopia: Further Analysis Using the 2011 Demographic and Health Survey

**DOI:** 10.1371/journal.pone.0130460

**Published:** 2015-06-19

**Authors:** Demewoz Haile, Yihunie Lakew

**Affiliations:** 1 Department of Public Health, College of Medicine and Health Sciences, Madawalabu University, Bale Robe, Ethiopia; 2 Ethiopian Public Health Association, Addis Ababa, Ethiopia; University College London, UNITED KINGDOM

## Abstract

**Background:**

Khat chewing has become a highly prevalent practice and a growing public health concern in Ethiopia. Although there have been many small scale studies, very limited national information has been available in the general population. This study aimed to identify factors associated with khat chewing practice among Ethiopian adults.

**Methods:**

The study used the 2011 Ethiopian demographic and health survey data. The survey was cross-sectional by design and used a multistage cluster sampling procedure. Bivariate and multivariable logistic regression models with adjusted odds ratio (AOR) and their 95% confidence intervals (CI) were used to quantify the predictors.

**Results:**

The overall khat chewing prevalence was 15.3% (95% CI: 14.90–15.71). Regional variation was observed with the highest in Harari [(53.2% (95% CI: 43.04–63.28)] and lowest in Tigray regional state [(1.1% (95% CI: 0.72–1.66)]. Multivariable analysis showed that Islam followers were 23.8 times more likely to chew khat as compared to Orthodox followers. Being a resident in Oromiya, South Nation, Nationalities and People (SNNP), Gambella, Harari and Dire Dawa regions had 1.9, 1.6, 3.1, 5.2 and 3.5 times higher odds of chewing khat as compared to Addis Ababa residents, respectively. Adults in the age group 45–49 years were 3.6 times more likely to chew khat as compared to 15–19 years. The middle and richest wealth quintiles were 1.3 and 1.5 times more likely to chew khat, respectively, as compared to the poorest category. Rural residents had 1.3 odds of chewing khat than urban residents. Those individuals who had occupation in sales, agriculture, service sector, skilled and unskilled manual workers were 1.6, 1.3, 2.4, 1.7 and 2.3 times more likely to chew khat, respectively, as compared to those who have no occupation. Females were 77% less likely to chew khat as compared to males. Formerly married and those experienced in child death had 1.4 and 1.2 times higher odds to chew khat as compared with those never married and never had child death, respectively. Those who attended mass media were 1.4 times more likely to chew khat compared with not attended.

**Conclusion:**

Khat chewing is a public health concern in Ethiopia. The highest wealth quintiles, older age group, rural residence, child death, formerly married, males, regions of Oromiya, SNNP, Gambella, Harari and Dire Dawa and Islamic followers had statistically significant association with khat chewing. Due attention needs to be given for these factors in any intervention procedures.

## Introduction

Khat was first discovered as CelastraceaeEdulisas plant by a Swedish botanist, Peter Forskal, during an expedition to Egypt and Yemen in 1761–1763d[1, 2]. Available literature showed that khat is a plant native to the Horn of Africa as early as in the 14^th^ century[3]. Historians agreed that khat use was observed in Ethiopia in 15^th^ century and the practice was transferred to the South-West of the Arabian Peninsula[1, 4].

Khat is a mildly narcotic substance, usually consumed by chewing the green leaf. The main psychoactive ingredients of khat includes cathine, cathinone and chemicals that are similar to amphetamines[5]. Khat is a strong stimulant that causes mild to moderate psychological dependence, although not as strong as that of alcohol and tobacco, and it can have serious health and economic consequence[6]. Regular khat chewing found to associate with elevated diastolic blood pressure among adults in Ethiopia[7]. It is significantly associated with higher risk of cardiogenic shock, stroke and death in patients presenting with acute coronary syndrome[8]. Regluar users of khat suffer from higher rates of mental distress[9]. Khat chewing is also associated with early initiation of sexual intercourse[10] and identified as a risk behaviour to HIV among taxi drivers[11]. Khat is found to associate with strain on family relationships, anti-social behaviour[12], insomnia[13, 14], anemia[15], as well as gastrointestinal disorders[16]. Hence, this psychoactive action has largely hampered the social, economic and health status of the society[12].

Despite of having such health and economic consequences, khat chewing has been practiced as stimulant and social custom for thousands of years in the Horn of Africa and Arabian Peninsula [17]. It persistently becomes a highly prevalent habit and a growing regional and international public health concern[18, 19]. Though the exact number of people who chew khat is unknown, the global estimates ranged from 5 to 10 million[20]. The largest number of chewers were found in Arabian Peninsula. As an example, half of the general adult population in Yemen consumes khat leaf on a daily basis[1, 21], of which 90% were adult males. Khat has also been widely consumed in East African countries including Somalia, Djibouti, Uganda, Kenya and Ethiopia [22, 23].

The number of khat chewers have significantly increased over the years in Ethiopia. Previously, khat was mainly cultivated and chewed in the eastern part of the country. Nowadays, evidence shows that khat is spreading to all Ethiopian geographic regions, religious and ethnic groups [14, 24, 25]. I commonly used as a stimulant in the academic environment and among long distance drivers [26–29], among Islamic followers [28, 29] and in certain population groups for a recreational substance [11].

Generic factors including normalization in the community [30], social mobility, accessibility (affordability and availability of khat leaf in the whole year) and the importance of khat as cash crop[31] have been identified as the major contributors to the widespread of khat chewing habit. However, very limited national studies have been available in the general community to identify contextual factors associated with khat chewing practice in order to influence policy and program interventions in Ethiopia. Therefore, this study aimed to identify factors associated with khat chewing practice among Ethiopian adults using 2011 Ethiopian national demographic and health survey data.

## Methods

### Ethical consideration

The data were downloaded and used after the purpose of the analysis was communicated and approved by Measure DHS. The original DHS data were collected in confirmation with international and national ethical guidelines. Ethical clearance for the original survey was provided by Ethiopian Public Health Institute Review Board, the National Research Ethics Review Committee (NRERC) at the Ministry of Science and Technology, the Institutional Review Board of ICF International, and the Center for Disease Prevention and Control (CDC).

### Data type and study design

This study used secondary data from the 2011 Ethiopian demographic and health survey (EDHS). Datasets from the adult men in the age group of 15–59 years and women in the age group15–49 years were used for the analysis. The original DHS data were collected using interview method in confirmation with international and national ethical guidelines. The EDHS was designed to provide socio-demographic and health indicators at national (urban and rural) and regional levels. The survey follows an international methodological approach and conducted in every five years interval. The 2011 EDHS samples were selected using a stratified, two-stage cluster sampling design. The detailed methodology is found elsewhere [6, 32]. Ethiopia is one of the sub-Saharan countries found in the Horn of Africa with 73.5 million populations based on the 2007 national population and housing census from which the sampling frame was derived[33].

### Data extraction

The adult men and women datasets were merged after understanding the detailed data coding and further data recoding was performed. A total of 30,625 adult populations were included in the analysis, and of which 14,110 were male population interviewed during the survey. Based on literature and availability of variables in the EDHS data: wealth index, age, occupation, child death experience, administrative region, religion, residence, education, exposure to mass media (indexed from reading newsletter, listening radio and television), gender and marital status were selected and extracted to be potentially associated with khat chewing. Besides to information on a wide-range of socio-demographic and economic variables, GPS points of EDHS clusters and khat chewing indicators were also extracted.

### Measurement of outcome variable

Khat chewing practice was assessed during the EDHS by asking the respondents about the number of days they chewed khat within a month. Khat chewing practices in the 30 days preceding the survey were used to measure the current prevalence of khat chewing habit.

### Data processing and analysis

Descriptive statistics including prevalence and frequency distributions were used to determine the level of khat chewing practice by socio-demographic characteristics. Bivariate analysis was used to show the association between socio-demographic characteristics and khat chewing practice. Variables that were determined statistically significant at p-value <0.25 during bivariate analysis were considered for adjustment in the level of multivariable logistic regression model [34, 35]. This cut-off point prevented removing variables that would potentially have an effect during multivariable analysis. A stepwise approach was used to assess the iteration of variables and to control potential confounders [36]. Sample weights were applied in order to compensate for the unequal probability of selection between the strata that has been geographically defined as well as for non-responses. A detailed explanation of the weighting procedure can be found in the DHS 2011[6]. The “svy” command in STATA version 11 (Stata Corporation, College Station, TX, USA) was used to weight the survey data. Furthermore, the GPS pointes were downloaded with permission from Measure DHS and merged with khat chewing prevalence in each study clusters. A map to show the distribution of khat chewing practice in all over the county is constructed.

## Results

The survey included un-weighted total adult populations of 30,625 in the age range from 15–59 years old. Among the respondents, 16,515(53.9%) were females. About 21,080 (68.8%) respondents were from rural areas. With regard to educational status, about 42% were not educated and 41% were under the category of primary education. The proportion of Christians (Orthodox, Protestant, and Catholic) was 60.7% followed by Islam 37.5%. The mean age of respondents was 29.0 with SD of 10.5.

As indicated in **Table 1**the overall prevalence of khat chewing in the 30 days preceding the survey was 15.3% (95% CI: 14.90–15.71). Regional variability was observed regarding khat chewing in Ethiopia. Khat chewing was highly prevalent in Harari regional state where above half of the population chew khat [53.2% (95% CI: 43.04–63.28)]. The prevalence in Dire Dawa was the second highest [44.9% (95% CI: 36.39–53.60)] followed by Oromiya [26.4% (95% CI: 25.59–27.21)] and Somali regional state [26.0% (95% CI: 22.53–29.55)]. The lowest prevalence was found in Tigray regional state [1.1% (95 CI: 0.72–1.66)]. The prevalence of khat chewing in rural places was higher [16.6% (95% CI: 16.13–17.08) as compared to urban areas [11.2% (95% CI: 10.48–11.95)]. The prevalence of khat chewing among adult men was higher [22.6% (95% CI: (21.92–23.30) as compared to females [9.1% (95% CI: (8.67–9.55)].

**Table 1:** Prevalence of khat chewing practice among adults across administrative region, residence and gender in Ethiopia, 2011.

Background characteristics	Total respondents	Weighted prevalence of khat chewing practice with 95%CI
**Administrative regions**		
Tigray	1,965	1.1(0.72–1.66)
Affar	256	18.1(13.62–23.03)
Amhara	8,344	7.8(7.24–8.39)
Oromiya	11,407	26.4(25.59–27.21)
Somali	598	26.0(22.53–29.55)
Benishangul-gumuz	323	7.7(5.18–11.05)
SNNP	5,760	8.2(7.51–8.92)
Gambela	132	14.9(9.78–22.04)
Harari	92	53.2(43.04–63.28)
Addis Ababa	1,621	11.7(10.22–13.36)
Dire Dawa	127	44.9(36.39–53.60)
**Residence**		
Urban	7,046	11.2(10.48–11.95)
Rural	23,579	16.6(16.13–17.08)
**Sex**		
Male	14,110	22.6(21.92–23.30)
Female	16,515	9.1(8.67–9.55)
**Total**	**30,625**	**15.3(14.90–15.71)**

**Fig 1:**
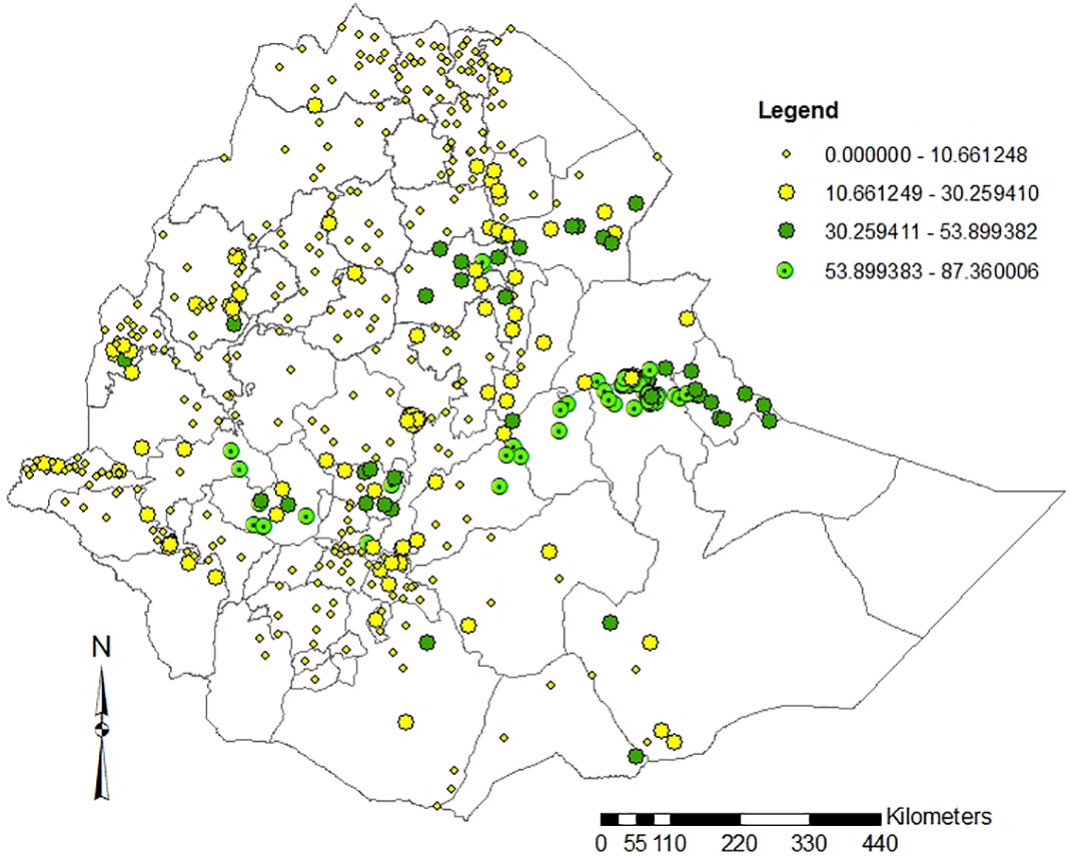
Map to show the distribution of khat chewing practice in Ethiopian Zones, 2011.

As indicated in **Fig** 1, the geo-reference distribution of khat in Ethiopia showed that higher concentration of khat chewing practice is located around eastern, central and north eastern parts of the country. The yellow dot in the map shows that khat is distributed throughout the country. There were no clusters included during EDHS 2011 from the extreme eastern parts of Ethiopia and that is the reason why an empty distribution has shown in the map.

In the multivariable logistic regression model, the highest wealth index quintile, older age group, Islam religion, administrative regions (Oromiya, SNNP, Gambella, Harari, Dire Dawa), occupations, rural residence, exposure to mass media, gender, formerly married and child death were found to have statistically significant association with chewing khat. Those found in middle and richest wealth quintile were 1.3 and 1.5 times at higher odds to chew khat as compared to poorest category, respectively. Those individuals who were in the 45–49 year category were 3.6 times more likely to chew khat as compared to individuals who were in the 15–19 years age category. Islam religion followers were 23.8 times more likely to chew khat as compared to Orthodox religion followers. Residents from Oromiya, SNNP, Gambella, Harari and Dire Dawa regionshad1.9, 1.6, 3.1, 5.2 and 3.5 times higher odds of chewing khat as compared to the Addis Ababa residents, respectively. Those individuals who had occupation in sales, agriculture, service sector, skilled and unskilled manual workers were 1.6, 1.3, 2.4, 1.7 and 2.3 times more likely to chew khat, respectively, as compared to those who have no occupation. The odds of khat chewing was 1.3 times higher among rural resident as compared to urban residents. Those individuals who had exposure to mass media had 1.4 times higher odds of chewing khat as compared to their counterparts. Females were 77% less likely to chew khat as compared to males. Formerly married (who had lost a spouse due to death or separation) and who had experienced at least one child death were 1.4 and 1.2 times more likely to chew khat as compared with those who had never married and never experienced child death(**Table 2**).

**Table 2:** Bivariate and multivariate logistic regression analysis showing factors associated with khat chewing practice among adult population in Ethiopia, 2011.

Variables	Unadjusted OR with 95%	Adjusted OR with 95%
**Wealth index**		
Poorest	Reference	Reference
Poor	1.1(1.035–1.276)*	1.10(0.96–1.269)
Middle	1.2(1.114–1.369)**	1.3(1.119–1.477)**
Rich	1.2(1.118–1.370)**	1.1(0.985–1.303)
Richest	0.91(0.825–1.010)	1.5 (1.236–1.851)**
**Age group**		
15–19	Reference	Reference
20–24	2.1(1.855–2.322)**	2.3(2.007–2.725)**
25–29	2.6(2.342–2.910)**	2.7(2.305–3.243)**
30–34	3.0(2.657–3.345)**	3.5(2.866–4.238)**
35–39	2.8(2.479–3.139)**	3.04(2.493–3.716)**
40–44	3.3(2.945–3.784)**	3.3(2.641–4.069)**
45–49	2.9(2.423–3.184)**	3.6(2.891–4.588)**
**Administrative regions**		
Addis Ababa	Reference	Reference
Tigray	0.1(0.055–0.135)**	0.2(0.090–0.245)**
Affar	1.7(1.168–2.364)**	0.4(0.269–0.635)**
Amhara	0.6(0.534-.753)**	0.8(0.656–1.074)
Oromiya	2.7(2.311–3.163)**	1.9(1.488–2.376)**
Somali	2.7(2.088–3.355)**	0.6(0.441-.811)**
Benishangul-Gumuz	0.6(0.407–0.973)	0.3(0.168–0.478)**
SNNP	0.7(0.561–0.801)**	1.6(1.232–2.068)**
Gambella	1.3(0.798–2.179)	3.1(1.590–5.891)**
Harari	8.6(5.542–13.261)**	5.2(2.958–9.298)**
Dire Dawa	6.1(4.191–8.980)**	3.5(2.104–5.682)**
**Occupation**		
Not working	Reference	Reference
Professional/technical/managerial	2.7(2.350–3.168) **	1.3(.963–1.819)
Clerical	2.0(1.614–2.500)*	1.1(.688–1.601)
Sales	2.4(2.205–2.687)**	1.6(1.419–1.889)**
Agriculture employee	2.3(2.079–2.445)**	1.3 (1.117–1.455)*
Services	3.0(2.429–3.615)**	2.4(1.574–3.575)**
Skilled manual	3.8(3.006–4.699)**	1.7(1.377–2.052)**
Unskilled manual	2.7(1.994–3.550)**	2.3 (1.573–3.353)**
**Experienced with child death**		
No	Reference	Reference
Yes	1.3(1.236–1.412)**	1.2(1.072–1.346)*
**Religion**		
Orthodox	Reference	Reference
Catholic	0.98(0.633–1.520)*	1.3(0.797–2.119)
Protestant	0.34(0.284–0.413)**	0.3(0.233–0.372)*
Islam	7.9(7.302–8.510)**	23.8(21.144–26.698)**
Traditional and others	2.0(1,540–2.699)**	1.6(1.179–2.223)**
**Residence**		
Urban	Reference	Reference
Rural	1.6(1.449–1.706)**	1.3(1.103–1.621)**
**Educational status**		
No formal education	Reference	Reference
Primary	1.1(1.028–1.171)*	0.92(0.830–1.024)
Secondary	1.2(1.094–1.340)**	0.89(0.722–1.090)
Higher	1.2(1.105–1.382)**	1.3(0.989–1.681)
**Exposed to mass-media**		
No	Reference	Reference
Yes	2.0(1.875–2.186)**	1.4(1.238–1.537)**
**Sex**		
Male	Reference	Reference
Female	0.08(0.064–0.096)**	0.23(0.207–0.258)**
**Marital status**		
Never married	Reference	Reference
Currently married	1.5(1.367–1.561)**	0.9 (0.789–1.042047)
Formerly married	1.2(1.059–1.347)	1.4(1.107–1.693)*

* P<0.05

**P<0.001

## Discussion

In Ethiopia, the overall prevalence of khat chewing was 15.3%, which is lower as compared to Yemen and Uganda[21, 23]. A small scale study conducted in Butajira, Ethiopia reported that lifetime experience of khat chewing was 55.7% and the prevalence of current use was 50% [37] which is higher than this national report. This large variation might be due to measurement and methodological differences. The health and demographic survey was conducted among a broad population and reported as an aggregate of very remote and urbanized areas in the country, which could explain why the findings differ from more local studies. A comparable prevalence of 15.9% and 17% were also reported from Addis Ababa and Dera District, Amhara regional state of Ethiopia, respectively [38, 39].

This study has shown that the highest prevalence of khat chewing was found in the central and eastern parts of Ethiopia as compared to northern part. In fact, central and especially eastern part of Ethiopia are the largest producer of khat for export as well as for national consumption[25]. Those residents of Oromiya, SNNP, Gambella, Harari and Dire Dawa region had higher odds of chewing khat as compared to the Addis Ababa residents. This could be due to the fact that Oromiya, Dire Dawa and Harari regions are the places where khat has been largely produced [25]which might affect the khat chewing practices of the population. Gambella and SNNP regions are also regions where khat production is spreading in these days. This implies that the availability of khat contributes to khat leaf consumption practices in the country.

This study identified a range of associated socio-economic factors. The higher wealth index was associated with chewing khat. Khat producers are usually in the rich group and have the chance to develop khat chewing habits while they cultivate and during business time. On the contrary, the poorest cannot afford to buy even essential consumable items and have less chance to chew khat due to lack of money. This is supported by a study that found having pocket money positively associated with khat chewing practice [40].

Being in the older age group category was positively associated with khat chewing. This is consistent with a study conducted in eastern Ethiopia[41]. Islam religion followers were at higher odds to chew khat as compared to Orthodox religion followers. This is again consistent with many studies done in Ethiopia [37, 38, 41–43]. Khat chewing is a common practice and traditionally accepted in Islam community that most people are chewing the leaf to gain maximum concentration during work and prayer time [44, 45].

This study found that rural residents were at higher odds to chew khat as compared to their urban counterparts. This contradicts other studies that have determined higher consumption among urban residents [38, 40]. The difference might be due to methodological approaches of studies and more than 80% Ethiopian population reside in rural places. Khat is also cultivated mostly in rural places of Ethiopia. Furthermore, our study is a national population based study which included both khat producing regions and others. This study found that females chew khat less likely as compared to males which is consistently agree with other studies [31, 38, 40, 41]. Khat chewing for female is more culturally restricted than males[43].

Khat chewing was also associated with type of occupation. In Ethiopia, khat is commonly consumed in certain population groups such as taxi and truck drivers who chew khat to stay awake during long driving times [46]. This study found that skilled and unskilled manual labour works, agriculture workers, sales and service provision workers and merchants were significantly khat chewers in which they might be used khat to gain energy and stimulant during work.

Exposure to mass media was significantly associated with increased odds of khat chewing. This result contradicts with other findings possibly due to the fact that most khat chewers read magazines and listen radio during the time of khat chewing. Therefore, exposure to mass media might be the outcome of khat chewing rather a predisposing factor. Child death and formerly married were associated with increased odds of khat chewing practice. This is possibly to stimulant themselves and avoid depression associated with the trauma of death and other social problems by using khat.

### Implications of the result

Khat as a psychoactive substance has largely hampered on the developments of social, economic and health status of the society in a nation [12]. Khat has adverse socio-economic consequences on many aspects of life including the loss of billions of hours of work[47] increased absenteeism, laziness and unpunctuality of employees on work[47, 48]. In Ethiopia, it was found that the negative effect of khat outweighs the money earn from the production of khat and can be a bottle neck for the fast economic growth in the country[31]. Khat production has not solved the problem of food security especially in khat producing districts[25] that khat has been cultivated at the expense of annual crop lands. The expansion of khat production has led to change in livestock composition due to reduced crop residues for fodder [5]. Khat chewing has also found to associate with lower academic performance among Ethiopian students [27]. The increasingly widespread of khat chewing habit increases the burden of non-communicable diseases and development of unhealthy risky behaviours in Ethiopia [10, 11, 16]. This study also supports that wealthier groups, farmers, and employees who were skilled manual and unskilled manual, in general the most productive age group were affected by khat. Reducing khat chewing habit in productive population groups could have an important effect on their health and in turn on economic development for the nation at large.

This was a secondary data analysis which missed important key variables such as peer pressure, perception about consequences and benefits of khat. The previous rounds of EDHS that conducted in 2000 and 2005 had no data on khat chewing indicators and as a result we couldn’t analyze and determine the increasing or decreasing scales of khat chewing practice across geographic regions and overtime. Although the authors understand that all khat chewing individuals do not consume the same amount or at the same frequency, the dosage of intake was missed in this study. However, despite such limitations it remains very important to have descriptive information on khat chewing and to identify predisposing factors associated with khat to draw greater attention in Ethiopia, a country in which khat was known to be historically originated.

## Conclusion

Khat chewing is practiced in all regions with the highest prevalence found in the eastern, central and north eastern parts of the country. The highest wealth index quintile, older age group, unskilled workforce, service and business workers, rural residents, exposure to mass media, experienced with child death, male sex, Islam religion followers and administrative regions (Oromiya, SNNP, Gambella, Harari and Dire Dawa)were factors statistically associated with khat chewing practice. Due attention should be given for those variables with higher odds and in places where higher distributions of khat chewing practice is found in eastern, central and north eastern parts of Ethiopia. Involving Islam religious leaders in prevention of khat chewing is also one of the recommended interventions. Appropriate policies and intervention procedures should be designed to curb khat chewing habits in Ethiopia and target these most at risk populations.
